# Immune Checkpoint Inhibitor-Induced Vogt–Koyanagi–Harada–like Disease Complicated by Inflammatory Macular Neovascularisation: A Case Report and Literature Review

**DOI:** 10.3390/diagnostics16162653

**Published:** 2026-08-20

**Authors:** Maria-Eleni Papavasileiou, Panagiotis Stavrakas, Petroula Mitri, Panteleimon Kalaitzakis, George Makris, Antonios Ragkousis

**Affiliations:** Uveitis Service, State Department of Ophthalmology, General Hospital of Athens “Georgios Gennimatas”, 11527 Athens, Greece; panos.stavrakas@yahoo.com (P.S.); mitripetroyla@gmail.com (P.M.); panteliskalaitzakis@yahoo.gr (P.K.); geomakopth@yahoo.gr (G.M.); tonyragou@gmail.com (A.R.)

**Keywords:** Vogt–Koyanagi–Harada (VKH)-like disease, immune checkpoint inhibitors (ICPI), immune-related adverse event (irAE), serous retinal detachment (SRD), bacillary layer detachment (BALAD), macular neovascularisation (MNV), choroidal neovascularisation (CNV)

## Abstract

**Background and Clinical Significance**: This paper presents a case of Vogt–Koyanagi–Harada (VKH)-like disease following nivolumab and ipilimumab therapy for squamous cell carcinoma of the lung, complicated by transient type 1 macular neovascularisation (MNV). **Case Presentation**: A 67-year-old man presented with reduced visual acuity, more pronounced in the left eye, accompanied by headache and neurosensory hearing loss for 10 days. He had been receiving combination therapy with nivolumab and ipilimumab for approximately nine weeks. Slit-lamp examination and multimodal imaging revealed multiple serous retinal detachments, choroidal folds, and bacillary layer detachment. A bilateral VKH-like syndrome was considered the most likely diagnosis, consistent with an immune-related adverse event (irAE). High-dose systemic corticosteroids were initiated, resulting in marked anatomical improvement and recovery of visual acuity. Following multidisciplinary discussion with the patient’s oncologist, ipilimumab was permanently discontinued and nivolumab was rechallenged in combination with chemotherapy after resolution of the ocular adverse events, given the progression of the underlying malignancy. Notably, optical coherence tomography angiography (OCTA) additionally demonstrated a type 1 non-exudative MNV, which resolved spontaneously during follow-up. **Conclusions**: Nivolumab and ipilimumab, targeting PD-1 and CTLA-4, respectively, are effective anticancer therapies but may induce immune-related adverse events involving the eye. VKH-like disease is a rare but potentially vision-threatening complication. Early recognition and prompt treatment are essential for favourable visual outcomes. In summary, this is a rare case of VKH-like disease associated with nivolumab and ipilimumab therapy, complicated by transient inflammatory type 1 MNV. Clear guidelines are needed regarding management of ocular immune-related adverse events and decisions on continuation or discontinuation of life-prolonging immunotherapy.

## 1. Introduction

Over the past few years, immune checkpoint inhibitors (ICPIs) have become integral in the treatment of metastatic cancers, including melanoma, lung, renal and bladder cancers. They act directly against key immune regulatory pathways, including programmed cell death-1 (PD-1) and cytotoxic T-lymphocyte antigen-4 (CTLA-4) [1]. The immune system relies on a balance between stimulatory and inhibitory signals to maintain immune homeostasis and prevent excessive inflammation or autoimmunity. CTLA-4 and PD-1 serve as major inhibitory checkpoints: PD-1 suppresses T-cell activation whereas CTLA-4 downregulates early T-cell activation during antigen presentation. By attacking these inhibitory pathways, ICPIs help the immune system to turn against cancer cells [2].

The combination of nivolumab (anti-PD-1) and ipilimumab (anti-CTLA-4) has recently been adopted as standard first-line treatment for the therapy of advanced non-small-cell lung carcinoma (NSCLC), combined with chemotherapy, as it offers superior overall survival and long-term clinical benefits [3]. Despite its significant clinical outcome, this therapeutic approach is associated with a wide range of immune-related adverse events (irAEs) attributable to immune system overactivation [4]. The incidence of irAEs increases dramatically to approximately 55% with combined anti-PD-1/CTLA-4 blockade [5]. These adverse events encompass a broad and clinically diverse spectrum, ranging from mild, self-limiting reactions to severe toxicities affecting the gastrointestinal, endocrine, pulmonary, cardiovascular, nervous and ocular systems. Their diagnosis is often challenging, requiring careful evaluation to differentiate immune-mediated toxicity from infection, disease progression, or paraneoplastic syndromes [6].

Ocular irAEs are infrequent, with uveitis representing the most common reported complication. Reported cases of these irAEs following immunotherapy include anterior and posterior uveitis, panuveitis and Vogt–Koyanagi–Harada (VKH)-like syndrome. Prompt diagnosis may not always be straightforward because the clinical findings may be subtle or nonspecific. For this reason, close patient monitoring is warranted, as the onset, severity, and clinical course may vary substantially among patients [7]. Of note, in our case, non-exudative macular neovascularisation was revealed in the course of the disease, which resolved spontaneously. Although MNV has previously been reported in association with ICPI-related VKH-like uveitis, the development of non-exudative type 1 MNV during the post-inflammatory recovery phase, with subsequent spontaneous regression without anti-VEGF therapy, represents an unusual clinical course [8].

## 2. Case Presentation

**Figure 1:**
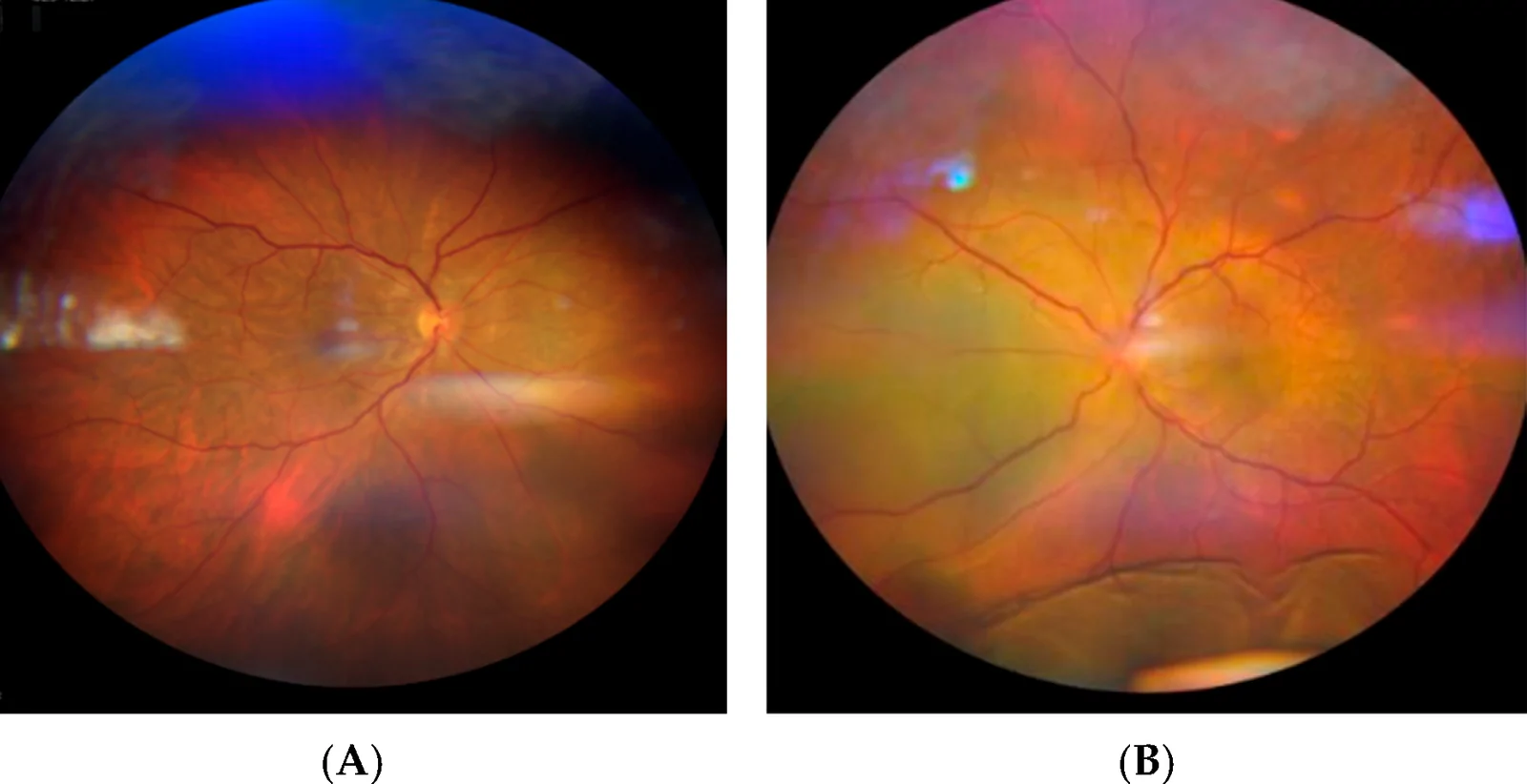
Wide-field color fundus photograph upon admission. (**A**) Incipient peripapillary/nasal/inferior serous retinal detachment of the right eye. (**B**) Nasal and inferior bullous serous retinal detachment of the left eye.

**Figure 2:**
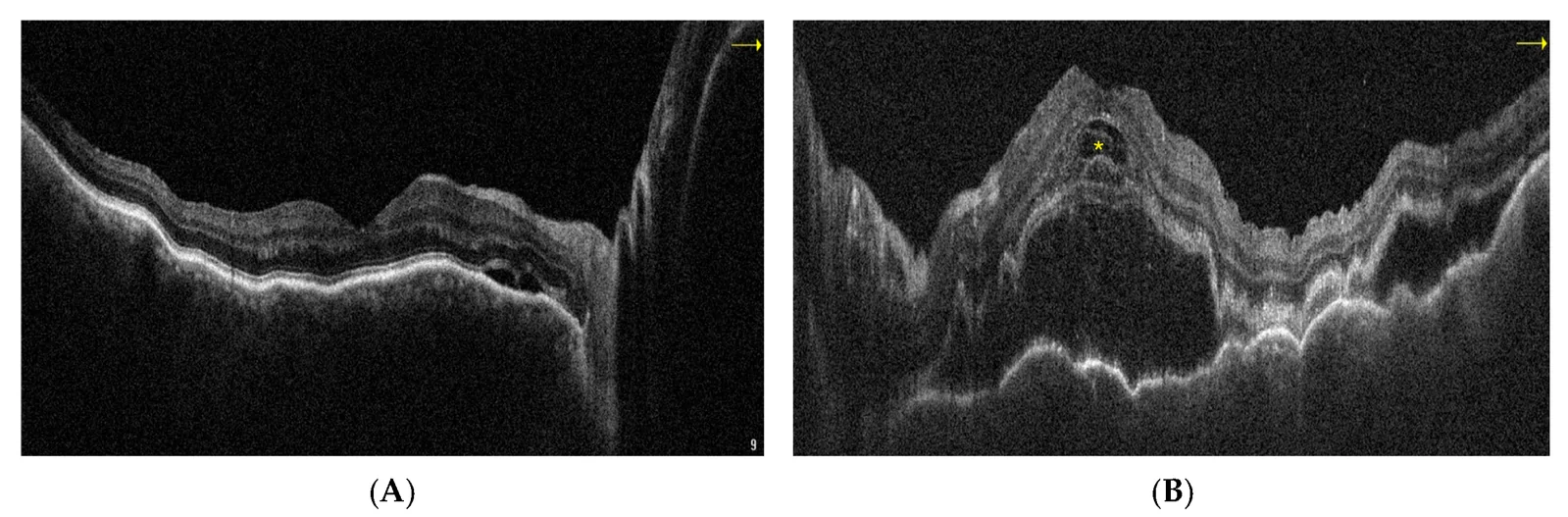
Optical coherence tomography (OCT) upon admission. (**A**) Subretinal fluid and choroidal folds in the right eye. (**B**) Serous retinal detachment, choroidal folds and bacillary layer detachment in the left eye (BALAD) (asterisk) *.

A 67-year-old man presented to our hospital with a known history of non-small-cell lung cancer (NSCLC). His medical history included dyslipidemia, benign prostatic hyperplasia, and a 100 p/y smoking history. The patient underwent imaging evaluation, including chest computed tomography (CT) scan, which revealed a 6.7 cm tumor in the right pulmonary hilum, exhibiting infiltrative extension and causing atelectasis in the lower medial segment of the right upper lobe. Concurrently, a positron emission tomography–computed tomography (PET-CT) scan demonstrated multiple secondary lymph node involvements across the cervical, supraclavicular, infraclavicular, mediastinal, and hilar regions, as well as a secondary metastatic lesion in the sternum. Magnetic resonance imaging (MRI) of the brain revealed no findings suspicious for secondary brain metastases. Subsequent endobronchial biopsy and histopathological examination revealed a non-keratinizing squamous cell carcinoma. The clinical stage was cT3N3M1, corresponding to stage IV disease. The patient received first-line treatment according to the CheckMate 9LA study, consisting of platinum-doublet chemotherapy with carboplatin (AUC 5) and paclitaxel (200 mg/m^2^) every 3 weeks, administered concomitantly with nivolumab (360 mg every 3 weeks) and ipilimumab (1 mg/kg every 6 weeks) [3]. Following four cycles of chemotherapy and immunotherapy, initiated on October 10 according to the treatment protocol described above, the patient developed visual symptoms on December 12, 13 days after the last treatment cycle. He presented to our clinic with progressively decreased visual acuity in his left eye, along with severe headache and neurosensory hearing loss. Best corrected visual acuity (BCVA) was 8/10 in the right eye (RE) and Counting Fingers (CF) In the left eye. Intraocular Pressure (IOP) was 10 and 9 mmHg, respectively. Slit-lamp examination revealed mild anterior uveitis in both eyes, with +1 anterior chamber cells and +1 vitreous cells in the RE and +3 anterior chamber cells and +1 vitreous cells in the LE. Dilated fundus examination revealed multiple serous retinal detachments (SRDs) in the LE and incipient peripapillary SRD in the RE. Ultra-widefield fundus images were acquired using a ZEISS CLARUS 500 (Carl Zeiss Meditec, Dublin, CA, USA, software version 1.2) (Figure 1). Optical coherence tomography (OCT) [RTVue XR Avanti System (Optovue, Inc., Fremont, CA, USA) with AngioVue software 2018.1.1.63], confirmed the SRDs, choroidal folds with corrugations of the RPE/choroid and bacillary layer detachment (BALAD) (Figure 2). Fluorescein angiography (FA) was performed at presentation, showing multiple leakage points and hot disc bilaterally. Indocyanine Green Angiography (ICGA) was performed after initiation of systemic corticosteroid therapy because immediate access to the imaging device was not available and was unremarkable. It is possible that the early anti-inflammatory effect of corticosteroid treatment may have attenuated the angiographic signs of active choroidal inflammation, contributing to the unremarkable ICGA findings. Wide-field fundus autofluorescence (FAF), both green-FAF and blue-FAF, showed a combined granular and diffuse pattern of hyperautofluorescence corresponding to the areas of SRD. ENT assessment, including pure-tone audiometry, confirmed neurosensory hearing loss, which had developed simultaneously with the ocular symptoms and headache. Two days after his admission to the hospital, visual acuity in his right eye deteriorated to 5/10, due to SRD involving the macula. A complete autoimmune and infectious work-up was performed, including full blood count, C-reactive protein (CRP), erythrocyte sedimentation rate (ESR), renal and liver function, serum angiotensin converting enzyme (sACE), interferon-gamma release assay (IGRA, QuantiFERON-TB Gold), fluorescent treponemal antibody absorption (FTA-Abs), venereal disease research laboratory (VDRL), chest X-ray and viral screening. All tests were unremarkable, except for a slight elevation in CRP (CRP = 9.8 mg/L normal limits <5 mg/L). A cerebrospinal fluid examination was not performed, as it was not deemed necessary by the neurologists. In addition, neuroimaging with magnetic resonance (MRI) was clear.

The combination of bilateral exudative retinal detachment, choroidal folds, and headache warranted consideration of several alternative diagnoses. Posterior scleritis was considered unlikely in the absence of ocular pain and characteristic ultrasonographic findings, including posterior scleral thickening, a T-sign, or sub-Tenon fluid. Uveal effusion syndrome was also considered; however, the patient’s normal axial length and absence of significant hyperopic refractive error argued against this diagnosis. Central serous chorioretinopathy or drug-associated serous retinopathy was considered less likely based on the simultaneous acute and extensive involvement of both eyes in an elderly patient, the choroidal folds, together with the concurrent inflammatory phenotype and subsequent resolution with steroid treatment. Choroidal metastatic or infiltrative disease was also considered given the patient’s underlying malignancy. However, the absence of focal choroidal masses or infiltrative thickening on multimodal imaging (B-scan and OCT; no signs of elevated choroidal lesion/mass) and lack of evidence of intraocular tumor infiltration argued against metastatic or infiltrative disease. Based on the patient’s history, multimodal imaging, exclusion of alternative infectious and inflammatory causes, and the temporal association with nivolumab/ipilimumab therapy, a diagnosis of ICPI-associated VKH-like disease was made. Although the patient did not represent idiopathic VKH, his presentation was compared with both the Revised Diagnostic Criteria and the 2021 SUN Classification Criteria for VKH (Table 1), supporting the diagnosis of VKH-like syndrome. Initially, intravenous methylprednisolone pulse therapy (1000 mg/day for three consecutive days) was administered, followed by oral methylprednisolone (64 mg, 0.8 mg/kg/day initial dose) together with topical dexamethasone eye drops administered six times daily in the left eye and three times daily in the right eye. Following clinical improvement after the first week of treatment, topical dexamethasone was tapered to once daily in the right eye for one week and then discontinued. In the left eye, the dosage was reduced to three times daily for one week, followed by a taper of one drop per week until discontinuation at week 3. Oral methylprednisolone was tapered by 8 mg every 7 days and maintained at 8 mg/day from week 10 onward.

One week after initiation of treatment, the patient showed significant anatomical and functional improvement, with near-complete resolution of the serous retinal detachments and gradual improvement in visual acuity. Additionally, the headache resolved and the patient’s hearing substantially improved.

**Figure 3:**
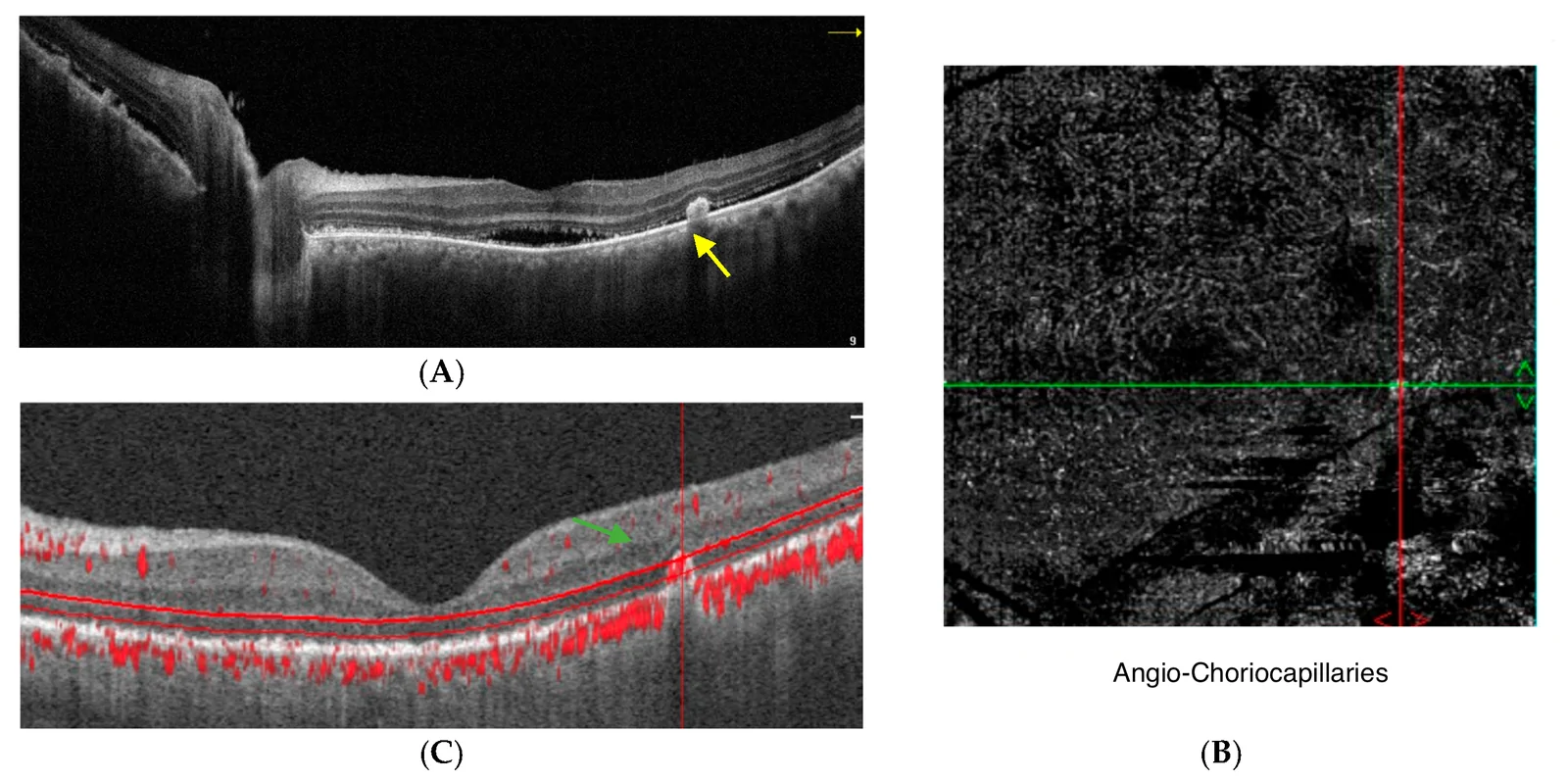
Left eye imaging 6 weeks after presentation (**A**): OCT: PED (yellow arrow) with high internal reflectivity, reduction in SRF and brush border pattern due to photoreceptors outer segments waste products at the outer surface of the detached neurosensory retina (**B**): OCTA en face image at the level of choriocapillaries. The vertical red and horizontal green lines indicate the corresponding location of the finding on the en face OCTA image (**C**): corresponding cross-sectional OCT image demonstrating flow signal inside the PED with the high internal reflectivity (green arrow).

Following communication with the patient’s oncologist and a discussion with the patient regarding the potential risks and benefits, a multidisciplinary approach was adopted. Following resolution of the ocular adverse events after corticosteroid treatment and taking into consideration the progression of the underlying disease, an individualized treatment decision was made for the patient, consisting of permanent discontinuation of ipilimumab and rechallenge with nivolumab in combination with chemotherapy. Six weeks later, visual acuity had improved to 10/10 and 5/10 in the right and left eye, respectively, with no clinical signs of active uveitis or subretinal fluid. Of note, an irregular pigment epithelial detachment (PED) with high internal hyperreflectivity was noted on OCT in the left eye (Figure 3).

OCT angiography (OCTA) was subsequently performed and demonstrated internal vascular flow within the lesion, confirming the diagnosis of inflammatory choroidal neovascularisation (iCNV). OCTA images were obtained using RTVue XR Avanti System (Optovue, Inc., Fremont, CA, USA) with AngioVue software (system’s software version: 2018.1.1.63). In addition to the SSADA algorithm, the system uses proprietary Motion Correction Technology (MCT) to reduce motion artifacts. The AngioVue software also provides quantitative OCTA measurements through AngioAnalytics, incorporating 3D Projection Artifact Removal (3D PAR). Three independent ophthalmologists, AR, GM and PS, reviewed the OCTA images and all agreed on the diagnosis of type I MNV. Because of lack of exudation in OCT such as intraretinal or subretinal fluid as well as the absence of retinal hemorrhages fundoscopically, no anti-VEGF was given at that time, and a regular follow-up was recommended for the patient in order to monitor the potential progression of the lesion.

**Figure 4:**
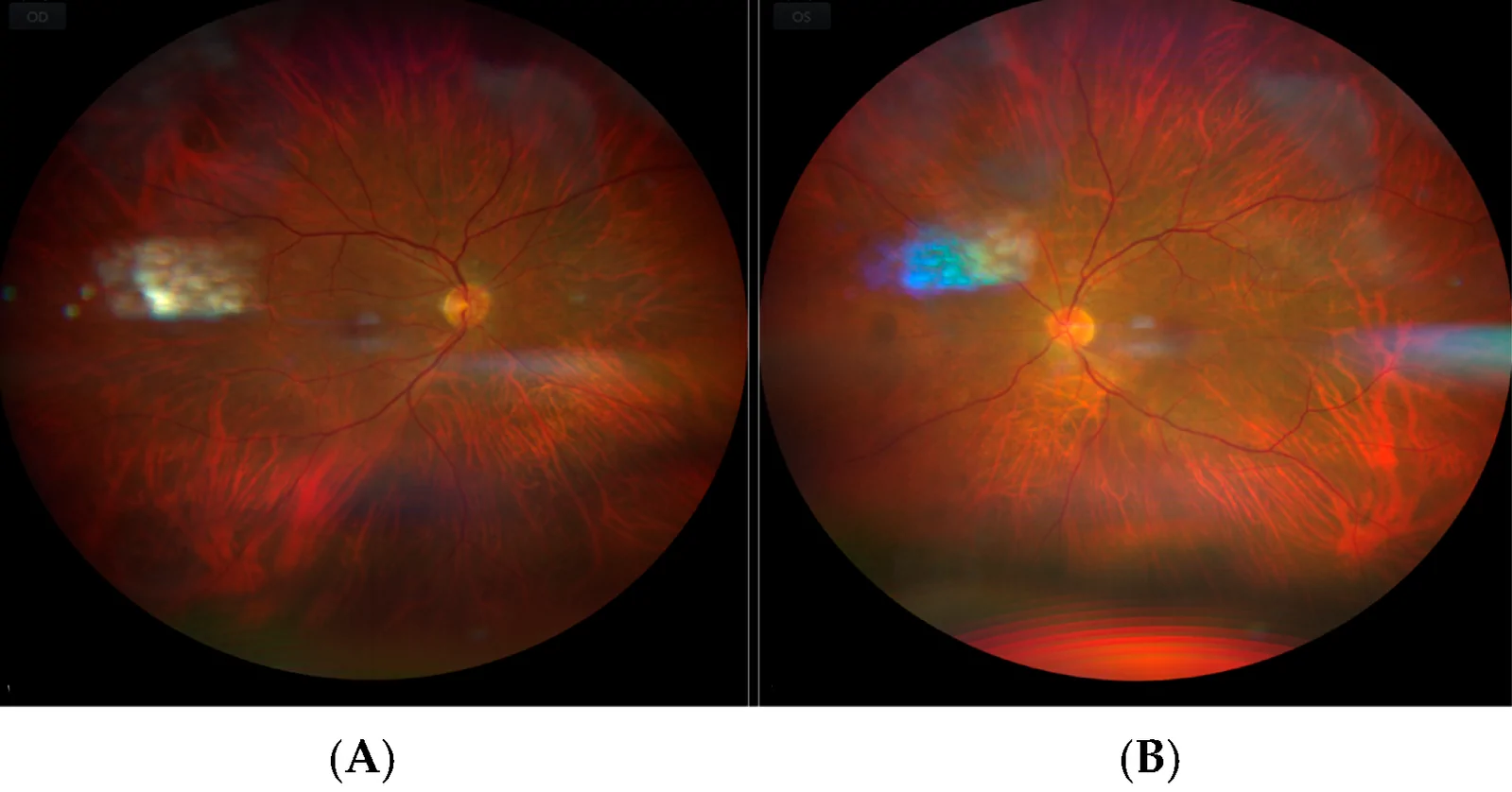
Wide-field color fundus photograph 10 weeks after presentation: (**A**) Resolution of peripapillary/nasal/inferior serous retinal detachment of the right eye. (**B**) Resolution of the nasal and inferior bullous serous retinal detachment of the left eye.

**Figure 5:**
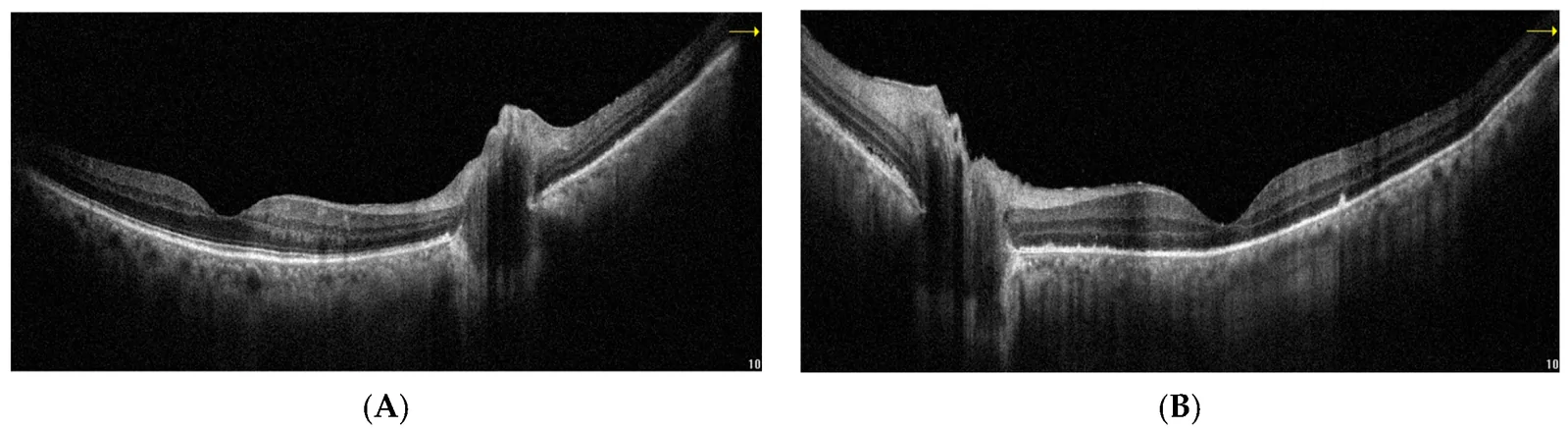
Optical coherence tomography 10 weeks after presentation: (**A**) Resolution of the SRF and choroidal folds in the right eye. (**B**) Resolution of SRD, choroidal folds and bacillary layer detachment with regression of MNV in the left eye.

**Figure 6:**
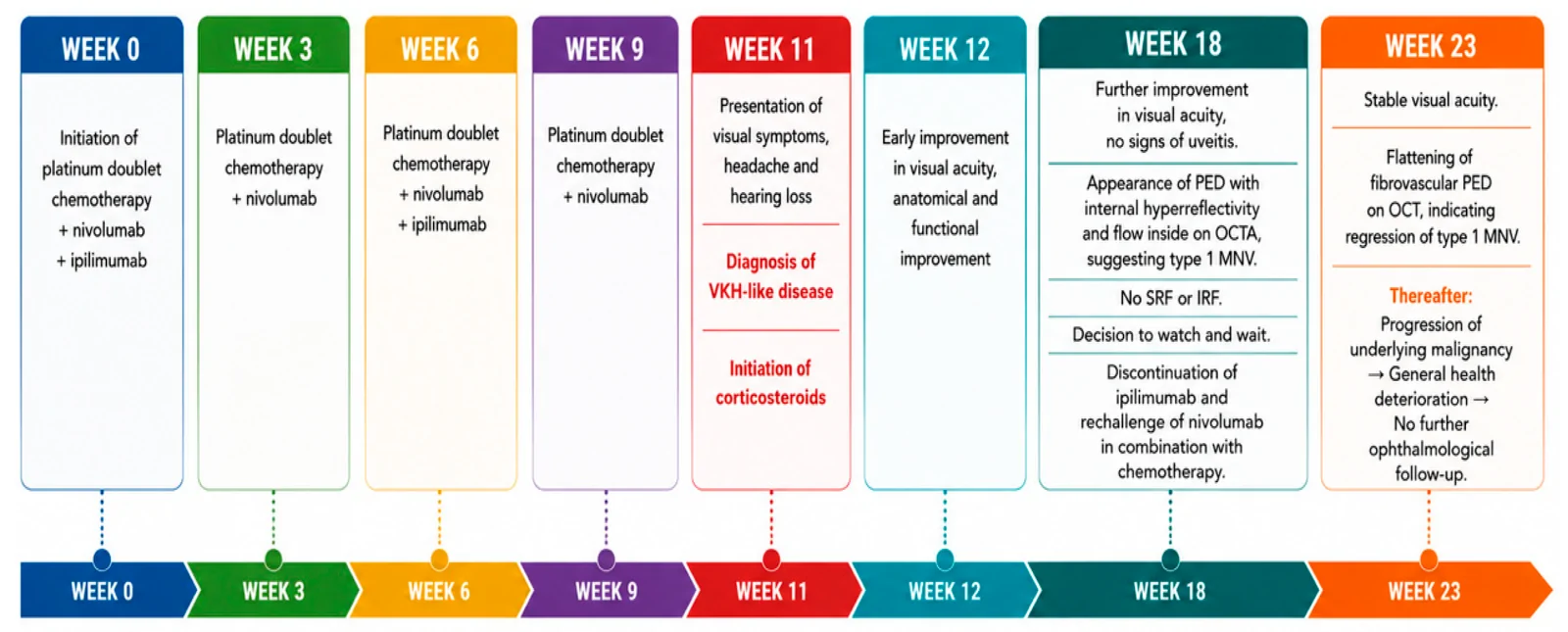
Timeline of the patient’s disease course and management.

One month later, the visual acuity was stable, retina was flat and the PED had flattened indicating that the iMNV spontaneously regressed (Figure 4 and Figure 5). Longer-term follow-up was not feasible because the patient’s general health deteriorated due to progression of his underlying malignancy. A comprehensive timeline of the patient’s disease course and management is provided in Figure 6.

## 3. Discussion

VKH disease is a bilateral granulomatous panuveitis with systemic manifestations, characterized by early and late ocular findings, with various clinical features depending on the stage of the disease, as described by the Revised Diagnostic Criteria of VKH disease [9,10]. These manifestations include auditory signs such as neurosensory hearing loss, tinnitus, vitiligo as well as neurological signs including headache, nausea and fever. Late findings, such as vitiligo, poliosis and alopecia, can appear in the course of the disease. The exact pathogenesis remains unclear; however, it is hypothesized that a T-cell-mediated autoimmune response targeting antigens related to melanocytes, melanin, and the retinal pigment epithelium (RPE) may play a key role in the disease process [11]. Our patient presented with ocular signs and systemic symptoms suggestive of VKH. In particular, he developed neurosensory hearing loss combined with severe headache and progressively decreased visual acuity approximately two and a half months after initiation of treatment with nivolumab and ipilimumab for squamous lung cell carcinoma, which is the standard first-line treatment for advanced non-small-cell lung cancer along with chemotherapy [12].

Immune checkpoint inhibitors (ICPIs) such as nivolumab and ipilimumab have demonstrated efficacy in the treatment of malignancies by enhancing T-cell activation, thereby promoting more effective elimination of tumor cells. More specifically, ipilimumab is an IgG1 human monoclonal antibody that leads to CTLA-4 inhibition. Cytotoxic T-lymphocyte antigen-4 (CTLA-4) is primarily expressed on activated lymphocytes and is simultaneously induced during T-cell-mediated cytotoxic responses. By blocking CTLA-4, T-cells remain active and can induce a strong immune response against cancer cells [13]. On the other hand, nivolumab is a fully human IgG4 monoclonal antibody that selectively binds with high affinity to programmed death-1 (PD-1) receptors [14]. PD-1 is found on T-cells and suppresses immune activity when it interacts with its ligands, PD-L1 and PD-L2, which are expressed on antigen-presenting cells (APCs). By blocking PD activity, nivolumab lifts abnormal immune inhibition, enabling immune cells to detect and attack tumor cells more effectively [15].

As the immune system is being activated by ICPIs, unintentional, serious immune-related adverse events (irAEs) can occur, involving almost every organ system [16]. These irAEs may affect the skin, where patients can develop rash, pruritus, or vitiligo; the gastrointestinal tract, leading to diarrhea and immune-mediated colitis; and the liver, resulting in hepatitis with elevated liver enzymes [17]. Endocrine dysfunction is also common and may involve thyroiditis, hypophysitis, adrenal insufficiency, or, more rarely, insulin-dependent diabetes mellitus [18]. Pulmonary toxicity can manifest as pneumonitis [19], while musculoskeletal involvement may present as arthralgia or inflammatory arthritis [20]. Less frequently, renal toxicity such as interstitial nephritis, cardiovascular complications including myocarditis, and neurological syndromes like neuropathies or encephalitis may occur [21]. When a combination therapy of ICPIs was administered, the incidence of these adverse events was reported to be higher [22].

Ocular irAEs induced by ICPIs are relatively rare and have been associated with a broad spectrum of manifestations including mild ocular surface disease, such as dry eye and conjunctivitis, to more severe inflammatory conditions including anterior, posterior, and panuveitis [23,24]. Additional less common but clinically significant complications include episcleritis, keratitis, retinal vasculitis, chorioretinitis, myositis and neuro-ophthalmic involvement such as optic neuritis and myasthenia gravis-related ocular symptoms. Among these adverse events, uveitis is by far the most common ocular complication (about 1% of patients) [23]. Anterior uveitis is the most frequently observed subtype overall, whereas posterior uveitis and panuveitis are less common, but they are typically associated with more severe inflammation and a greater risk of vision loss [25].

However, it is worth mentioning that other categories of anticancer agents widely used in oncology can also cause ocular adverse effects. Characteristically, MEK and BRAF inhibitors can cause target-mediated toxicity by blocking the mitogen-activated protein kinase signaling pathway [26]. ΜΕΚ inhibitors (like trametinib, cobimetinib, or binimetinib) typically cause a central serous chorioretinopathy (CSCR)-like phenotype, known as MEK-associated retinopathy (MEKAR) [27]. BRAF inhibitors (such as dabrafenib, vemurafenib and encorafenib) have been implicated in mixed target- and immune-mediated ocular toxicity. BRAF inhibitors as monotherapy and BRAF/MEK combination have been associated with uveitis, VKH-like disease and retinal vein occlusion [28]. FGFR inhibitors (erdafitinib, pemigatinib and futibatinib) can also cause serous retinopathy similar to MEKAR. For MEKAR or FGFR-associated retinopathy, observation along with drug withdrawal is the typical approach, whereas for BRAF or ICPI immune-mediated uveitis the recommended course of action is to start with topical steroids escalating to periocular or systemic steroids when needed depending on uveitis severity, following a stepwise strategy [27,28,29]. A different mechanism of ocular toxicity is the one associated with antibody–drug conjugates (ADCs), such as belantamab mafodotin used in multiple myeloma treatment have been associated with distinct ocular manifestations, particularly corneal epithelial changes [30].

Regarding previously reported cases of ICPI-associated VKH-like disease, a comprehensive literature search was conducted in PubMed/MEDLINE to identify published cases. The search was performed using combinations of the following terms: (“Vogt-Koyanagi-Harada” OR “VKH” OR “VKH-like” OR “Harada” OR “serous retinal detachment” OR “exudative retinal detachment” OR “choroidal detachment”) AND (“immune checkpoint inhibitor” OR “immune checkpoint inhibitors” OR “immunotherapy” OR “immune-related adverse event” OR “nivolumab” OR “ipilimumab”). Additional relevant publications were identified by reviewing the reference lists of retrieved articles and relevant review papers. The search included publications available through June 2026. Cases were included when a VKH or VKH-like ocular phenotype was described in temporal association with ICPI therapy. Cases of isolated anterior uveitis or nonspecific ICPI-associated ocular inflammation without a VKH-like phenotype were excluded.

A total of 32 cases were identified [Table 2; [8,24,31,32,33,34,35,36,37,38,39,40,41,42,43,44,45,46,47,48,49,50,51,52,53,54,55,56,57]]. Most of these patients had melanoma (20/32, 62.5%). Eleven (34.3%) received nivolumab alone, seven (21.8%) received pembrolizumab alone, six (28.7%) received nivolumab/ipilimumab combination therapy, and three (9.4%) received tislelizumab alone, while the remaining cases involved treatment with ipilimumab monotherapy, cemiplimab, atezolizumab, or combination immune checkpoint inhibitor regimens. Twenty-four (75%) received systemic corticosteroid therapy, and twenty-two of these had partial or complete resolution of uveitis and/or visual acuity, while the remaining two patients experienced a recurrence of uveitis, which subsequently resolved during follow-up. Regarding these two cases, ICPI therapy was continued in one of the two patients despite development of VKH-like uveitis [28]. Regarding oncological outcomes, ten cases (31.2%) showed complete or partial responses. Concerning neovascular complications, only one case of CNV with SRF/IRF was reported in the published literature, in a patient described by Qian et al. [8]; however, the CNV subtype, specific CNV treatment, and final visual acuity were not reported.

Only a few published reports exist of ipilimumab- and nivolumab-associated VKH-like disease. This is of the highest importance, since dual immunotherapy plus chemotherapy is currently the first-line treatment for patients with metastatic NSCLC, providing long-term survival benefit, and there should be a low threshold of clinical suspicion for irAEs among oncologists, pulmonologists, and ophthalmologists for potential ocular complications [3]. The occurrence of ocular irAEs could potentially alter the therapeutic approach of these patients and could lead to different treatment options [58].

According to previous research findings, the mean time to onset of uveitis was 22.2 ± 29.6 weeks after immunotherapy; in our case, visual symptoms developed almost 11 weeks after initiation of immunotherapy [59]. Based on the American Society of Clinical Oncology Practice Guidelines (ASCO) for the management of irAEs in patients treated with ICPI therapy, our case is classified as grade 4 presentation and should be managed with permanent discontinuation of the ICPI, urgent ophthalmological evaluation and corticosteroids administration (systemic and intravitreal or ocular or topical administration) [60]. Taking into consideration these guidelines and the severity of the adverse events, a multidisciplinary approach was adopted in collaboration with the patient’s oncologist. Following resolution of the ocular adverse events after corticosteroid treatment and taking into consideration the progression of the underlying disease, an individualized treatment decision was made for the patient, consisting of permanent discontinuation of ipilimumab and rechallenge with nivolumab in combination with chemotherapy. In this context, the balance between the benefits of cancer treatment and the risk of treatment-related ocular adverse events had to be carefully considered. After corticosteroid treatment, the patient showed resolution of symptoms, improvement in visual acuity, reduction in inflammation and retinal flattening. Nevertheless, long-term monitoring was not feasible because the patient’s general health deteriorated due to progression of his underlying malignancy. This case underscores the importance of developing standardized approaches to ocular immune-related adverse events while allowing for individualized treatment decisions. As ICPIs may provide substantial oncological benefit, determining whether to discontinue, modify, or resume immunotherapy in the setting of ocular inflammation remains challenging and should involve careful multidisciplinary evaluation.

Although MNV has previously been reported in association with ICPI-related VKH-like uveitis, the development of non-exudative type 1 MNV during the post-inflammatory recovery phase, with subsequent spontaneous regression without anti-VEGF therapy, represents an unusual clinical course. Although iMNV is a known complication that happens in approximately 10% of VKH cases, this is the second case report of iMNV in VKH-like disease secondary to ICPI immunotherapy [8]. At this point, it is important to emphasize that the differential diagnosis between iMNV and an incidental age-related vascularized PED is also of particular importance. In AMD, type 1 MNV commonly occurs in the context of an underlying degenerative phenotype, with associated drusen, pigmentary RPE abnormalities, RPE irregularity or atrophy, and, in some cases, characteristic choroidal alterations. Although choroidal biomarkers such as choroidal thickness, choroidal vascularity index, choroidal contour, choroidal vessel thickness analysis and choroidal volume may demonstrate characteristic alterations across AMD phenotypes, these parameters are not disease-specific; in our patient, the de novo development of the fibrovascular PED after the inflammatory episode provided stronger evidence against an incidental age-related MNV [61]. Type 1 MNV following posterior uveitis can occur by two pathophysiological mechanisms. The first one is by inflammation of the retina and choroid, which impairs blood flow, leading to retina and choroidal hypoxia, that stimulates neovascularisation beneath or within the retina [62]. The second mechanism is by inflammatory mediated disruption of retinal pigment epithelium (RPE)–Bruch’s membrane complex, which leads to break down of the outer blood–retinal barrier, allowing new blood vessels to grow upward from the choroid [63,64]. While MNV is considered uncommon in this particular type of uveitis, early recognition and prompt management are crucial to prevent vision loss, although in some cases, like our patient, the neovascularisation may be non-exudative, self-limited and resolve spontaneously [65]. Although evidence-based guidelines for non-exudative iMNV are lacking, emerging evidence suggests that close observation may be an appropriate strategy in the absence of clinical or anatomical signs of activity. In particular, Arora et al. reported patients with iCNV without intraretinal or subretinal fluid who were managed with observation and close follow-up without anti-VEGF therapy [66]. Similarly, Sacconi et al. emphasized the importance of close monitoring of non-exudative MNV, while Berni et al. concluded that prophylactic treatment is not currently recommended because of the lack of prospective evidence supporting this approach [67,68]. Careful monitoring and multimodal imaging are crucial to prevent vision-threatening complications and avoid unnecessary interventions [69].

## 4. Conclusions

In summary, this is a rare case of VKH-like uveitis following combination treatment of nivolumab and ipilimumab for squamous cell carcinoma of the lung, which was complicated by transient type 1 inflammatory macular neovascularisation. This case highlights the need for clear guidelines on managing complications and for adjusting dosing and treatment strategies in the context of emerging immunotherapies. Given that immunotherapies for cancer are often critical for patient survival, decisions regarding treatment discontinuation following ocular inflammation are particularly complex. Management of ICPI-associated uveitis generally follows a stepwise approach, with treatment escalating from topical corticosteroids to local or systemic corticosteroids and, if required, additional immunosuppressive agents based on the type and severity of uveitis. Determining whether to cease therapy and establishing appropriate thresholds based on the severity of ocular involvement remains a significant clinical challenge. These considerations underscore the need for larger, future studies to better inform evidence-based guidelines.

## Figures and Tables

**Table 1:** Comparison of the patient’s findings with the Revised Diagnostic Criteria (RDC) and the 2021 SUN Classification Criteria for Vogt–Koyanagi–Harada (VKH) disease.

Criterion	Revised Diagnostic Criteria (2001)	2021 SUN Classification Criteria	Present in Our Patient	Comments
No history of penetrating ocular trauma or surgery	Required	Required	**✓**	No history of ocular trauma or surgery
Alternative ocular disease excluded	Required	Required	**✓**	Infectious, autoimmune and neoplastic investigations were negative
Bilateral ocular involvement	Required	Required	**✓**	Bilateral panuveitis with serous retinal detachments
Multifocal serous retinal detachments	Included	Included	**✓**	Present in both eyes (left predominance initially)
Diffuse choroiditis/choroidal thickening	Included	Included	**✓**	Choroidal thickening and folds on OCT
FA findings consistent with VKH	Supportive	Included	**✓**	Multiple pinpoint leakage and optic disc hyperfluorescence
ICGA abnormalities	Supportive (not mandatory)	Not mandatory	**✗**	ICGA was performed on week after treatment initiation and was unremarkable
Neurological manifestations	Supportive	Included when present	**✓**	Severe headache
Auditory manifestations	Supportive	Included when present	**✓**	Sensorineural hearing loss
Integumentary findings (vitiligo, poliosis, alopecia)	Required only for complete VKH	Not required	**✗**	Absent during follow-up
Response to corticosteroids	Not diagnostic criterion	Not diagnostic criterion	**✓**	Rapid anatomical and functional improvement

**Table 2:** Reported cases of ICPI-associated VKH-like disease.

Author and Date	Cancer	ICPI	VKH-like Ocular Presentation	Treatment	Visual Outcome	Recurrence	CNV/MNV	ICPI Discontinued?	Neoplastic Outcome
Crosson et al., 2015 [31]	Melanoma	Ipilimumab	VKH-like uveitis	Surveillance	Improved/resolved	NR	NR	Yes	NR
Fierz et al., 2016 [32]	Melanoma	Ipilimumab	VKH-like uveitis	Topical prednisolone and PO prednisolone	Improved/resolved	NR	NR	Yes	Complete remission
Arai et al., 2017 [33]	Metastatic melanoma	Nivolumab	VKH-like uveitis	Topical corticosteroids + mydriatics	Resolution	No	NR	No	Stable
Bricout et al., 2017 [34]	Melanoma	Pembrolizumab	Bilateral exudative retinal/choroidal detachment	Topical dexamethasone and subconjunctival betamethasone injection	NR	NR	NR	NR	Partial remission
Conrady et al., 2018 [35]	Melanoma	Pembrolizumab	VKH-like posterior uveitis	IV, PO, topical corticosteroids	Improved	NR	NR	NR	NR
Fujimura et al., 2018 [36]	Metastatic Melanoma	Nivolumab, dabrafenib and trametinib	VKH-like uveitis	PO prednisone and IV methylprednisolone	Partial remission	No	NR	ICPI re-challenge	Partial remission
Tamura et al., 2018 [37]	NSCLC	Pembrolizumab	VKH-like uveitis with serous retinal detachment	Corticosteroid, unspecified	Improved/resolved	NR	NR	Yes	Death
Obata et al., 2019 [38]	Melanoma	Nivolumab	VKH-like uveitis with serous retinal detachment	Topical betamethasone, tropicamide, phenylephrine	Partial resolution	NR	NR	Yes	Progression
Dolaghan et al., 2019 [39]	Melanoma	Pembrolizumab	VKH-like uveitis with exudative retinal detachment	PO steroids and topical prednisolone acetate	Improved	No	NR	No	NR
Reid et al., 2019 [40]	Melanoma	Pembrolizumab	VKH-like uveitis	IV methylprednisolone, peribulbar triamcinolone acetonide, OVD injections in the AC	Improved/resolved	NR	NR	NR	Complete remission
Gambichler et al., 2020 [41]	Melanoma	Nivolumab	VKH-like uveitis	IV methylprednisolone, then PO prednisolone	Improved	NR	NR	NR	NR
Enomoto et al., 2021 [42]	Melanoma	Pembrolizumab	VKH-like uveitis	PO prednisone and topical betamethasone	Partial resolution	NR	NR	Yes	NR
Minami et al., 2021 [43]	Melanoma	Nivolumab + ipilimumab	VKH-like panuveitis	Sub-Tenon triamcinolone, topical betamethasone	Resolution	NR	NR	ICPI re-challenge	Disease progression
Godse et al., 2021 [44]	Melanoma	Nivolumab + ipilimumab	VKH-like uveitis	PO prednisolone and MMF	Resolution	NR	NR	Yes	Complete remission
Stürmer et al., 2021 [45]	Melanoma	Nivolumab + ipilimumab	VKH-like panuveitis	Topical prednisolone acetate, PO prednisolone	Resolution	NR	NR	Yes	Partial remission
Suwa et al., 202 2 [46]	NSCLC	Atezolizumab	VKH-like uveitis with serous retinal detachment	IV methylprednisolone, PO prednisolone and topical steroids	Resolution	NR	NR	Yes	NR
Hwang et al., 2022 [47]	Ovarian cancer	Nivolumab	VKH-like uveitis	IV/oral corticosteroids	Recurrence	No	NR	No	Stable
Chaudot et al., 2022 [24]	Melanoma	Nivolumab + ipilimumab	VKH-like panuveitis	IV, PO, topical steroids	Resolution/improved	NR	NR	No	Partial remission
Nagai et al., 2023 [48]	Gastric cancer	Nivolumab	VKH-like uveitis	Topical/sub-Tenon ± systemic corticosteroids	Partial resolution	No	NR	Yes	NR
Huang et al., 2023 [49]	Squamous cell carcinoma/Melanoma	Cemiplimab	VKH-like uveitis	PO prednisone, azathioprine, MME, ADA, sub-Tenon triamcinolone	Recurrence/partial resolution	Yes	NR	Yes	NR
Pole et al., 2023 [50]	Melanoma	Nivolumab	VKH-like uveitis	IV corticosteroids	Partial resolution	NR	NR	Yes	NR
Madoe et al., 2023 [51]	Melanoma	Nivolumab	VKH-like uveitis with serous retinal detachment/choroidal involvement	Topical dexamethasone, PO methylprednisolone	Partial resolution	NR	NR	Yes	Partial remission
Ng et al., 2023 [52]	Clear cell RCC	Nivolumab	VKH-like disease with subsequent chorioretinal complications	PO prednisone	Resolution	NR	NR	NR	NR
Tieger et al., 2023 [53]	RCC	Nivolumab	VKH-like uveitis	Oral corticosteroids	Resolution	NR	NR	NR	NR
Qian et al., 2024 [8]	RCC	Nivolumab	VKH-like panuveitis	Topical/oral corticosteroids ± infliximab	Resolution	NR	**CNV reported**	Yes	NR
Qian et al., 2024 [8]	SCLC	Nivolumab	VKH-like uveitis	Topical/oral corticosteroids	Resolution	NR	NR	Yes	NR
Qian et al., 2024 [8]	Melanoma	Nivolumab + ipilimumab	VKH-like uveitis	Topical/oral corticosteroids	Resolution	NR	NR	Yes	Complete remission
Denu et al., 2024 [54]	GEJ adenocarcinoma	Pembrolizumab	Harada/VKH-like uveitis	Local + systemic corticosteroids	Returned to baseline	NR	NR	Yes	Complete remission
Ding et al., 2025 [55]	1. Metastatic endometrial adenocarcinoma 2. SCLC	Tislelizumab	VKH-like/panuveitic phenotype	Topical corticosteroids-transcutaneous triamcinolone	Improved	NR	NR	NR	NR
Zhou et al., 2026 [56]	Esophageal cancer	Tislelizumab	VKH-like uveitis with optic-disc edema and exudative retinal detachment	Systemic corticosteroid pulse	Recurrence/partial resolution	Yes	NR	IPCI rechallenge	NR
Villain et al., 2026 [57]	Metastatic melanoma	Nivolumab + ipilimumab	Severe VKH-like syndrome with persistent choroidal detachment	Corticosteroids → surgical drainage	Improved after surgery	NR	NR	Yes	Control

Abbreviations: ICPI, immune checkpoint inhibitor; VKH, Vogt–Koyanagi–Harada; CNV, choroidal neovascularization; MNV, macular neovascularization; NSCLC, non-small-cell lung cancer; RCC, renal cell carcinoma; SCLC, small-cell lung cancer; GEJ, gastroesophageal junction; NR, not reported.

## Data Availability

The data supporting the findings of this study are available within the manuscript. Any additional data can be provided upon reasonable request.
